# Metabolic Proximity in the Order of Colonization of a Microbial Community

**DOI:** 10.1371/journal.pone.0077617

**Published:** 2013-10-30

**Authors:** Varun Mazumdar, Salomon Amar, Daniel Segrè

**Affiliations:** 1 Bioinformatics Program, Boston University, Boston, Massachusetts, United States of America; 2 Center for Anti-Inflammatory Therapeutics; Boston University Goldman School of Dental Medicine, Boston, Massachusetts, United States of America; 3 Department of Biology and Department of Biomedical Engineering, Boston University, Boston, Massachusetts, United States of America; University of Oklahoma Health Sciences Center, United States of America

## Abstract

Microbial biofilms are often composed of multiple bacterial species that accumulate by adhering to a surface and to each other. Biofilms can be resistant to antibiotics and physical stresses, posing unresolved challenges in the fight against infectious diseases. It has been suggested that early colonizers of certain biofilms could cause local environmental changes, favoring the aggregation of subsequent organisms. Here we ask whether the enzyme content of different microbes in a well-characterized dental biofilm can be used to predict their order of colonization. We define a metabolic distance between different species, based on the overlap in their enzyme content. We next use this metric to quantify the average metabolic distance between neighboring organisms in the biofilm. We find that this distance is significantly smaller than the one observed for a random choice of prokaryotes, probably reflecting the environmental constraints on metabolic function of the community. More surprisingly, this metabolic metric is able to discriminate between observed and randomized orders of colonization of the biofilm, with the observed orders displaying smaller metabolic distance than randomized ones. By complementing these results with the analysis of individual vs. joint metabolic networks, we find that the tendency towards minimal metabolic distance may be counter-balanced by a propensity to pair organisms with maximal joint potential for synergistic interactions. The trade-off between these two tendencies may create a “sweet spot” of optimal inter-organism distance, with possible broad implications for our understanding of microbial community organization.

## Introduction

In many natural environments, bacteria and other micro-organisms are part of spatially structured ecosystems, and engage in complex interactions, involving the exchange of nutrients and chemical signals [1], [2]. Such communities provide their members with protection from environmental perturbations, and allow for effective utilization of available resources. Modifications of the environment, such as a change in diet in a human host, can cause shifts in the composition of a microbial community. In turn, the collective metabolic activity of the community itself can substantially modify the environment, and set the stage for transitions between health and disease states. While 16S rRNA studies [3] and metagenomic DNA sequencing [4] have been very helpful in providing provide global snapshots of the composition and biological functions of a community, a big gap still exists in our understanding of the forces that shape specific interactions between different organisms. Systems biology approaches have started to provide valuable insight into the metabolic basis of interactions between different species in elementary [5] and complex [6] microbial ecosystems. However, a lot is still unknown on how properties of individual species give rise to global ecosystem organization.

**Figure 1:**
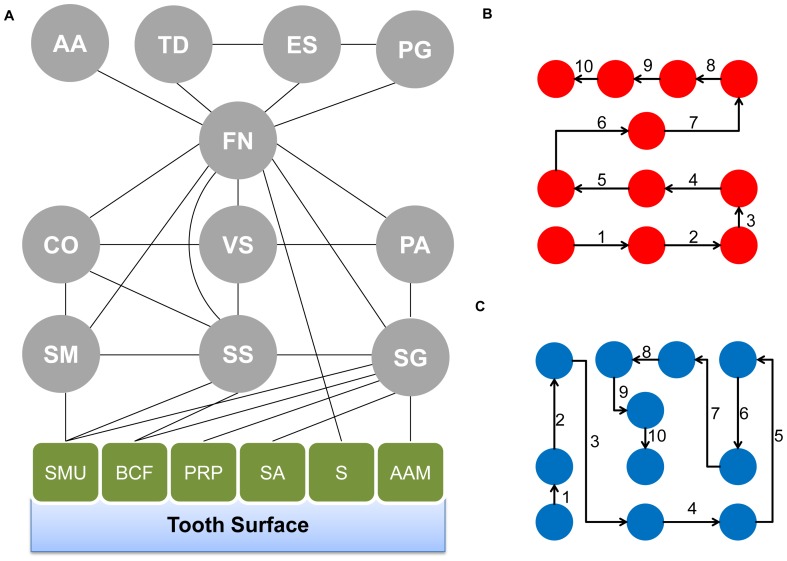
A simplified model of dental biofilm. (A) Rectangular nodes represent components of the salivary pellicle while circular nodes represent organisms in the biofilm. Lines represent known interactions (often mediated by adhesin molecules) between different components of the biofilm. The organism abbreviations are as follows: Layer 1: SM-*Streptococcus mitis*, SS-*Streptococcus sanguinis* and SG-*Streptococcus gordonii*. Layer 2: CO-*Capnocytophaga ochracea*,VS-*Veionella* (represented by *Veillonella parvula*), and PA-*Propionibacterium acnes*. Layer 3: FN-*Fusobacterium nucleatum*. Layer 4: AA-*Aggregatibacter actinomycetemcomitans*, TD-*Treponema denticola*, ES-*Eubacterium* (represented by *Eubacterium eligens*), and PG-*Porphyromonas gingivalis*. The salivary receptors have the following abbreviations: SMU-Sialylated mucins, BCF-Bacterial cell fragment, PRP-Proline rich protein, SA-Salivary agglutinin, S- Statherin and AAM-Alpha amylase. (B) A schematic representation of one of the many possible step-wise orders of colonization that conforms to the layered organization inferred from the literature, i.e. is such that the path that walks through the different species is monotonically departing from the salivary pellicle upwards. In our calculations of inter-species metabolic distances, we average the distances between any two species connected by a segment. This calculation is performed for all paths that reflect the order of colonization, giving rise to the distributions shown in Fig. 2. (C) A schematic representation of one of the many possible randomized orders of colonization that do not follow the order of the literature-derived layers; for the 11 organisms present in the biofilm there are 11! possible permutations.

Here, we address this problem by presenting an intermediate-scale approach to elucidate the role of metabolism in determining the order of colonization in a microbial community. Our approach captures the complexity of how eleven species spatially organize in a biofilm, by using a mathematical description of metabolism that lies in between the detailed quantitative power of stoichiometric models [7], and the coarse enrichment analyses typically obtained from metagenomic studies [4], [8]–[11]. We focus specifically on one of the most intensively characterized biofilm systems, the dental biofilm, which plays a crucial role in tooth and gum diseases. Oral pathogens such as *Porphyromonas gingivalis* are also known to be able to enter the blood stream, possibly causing cardiovascular disease. The collection of bacteria present on the surface of teeth, anchored to the salivary pellicle, has a specific spatial structure, which has been mapped and investigated in detail [12]–[14]. The biofilm structure is made up of a number of different species, which aggregate together over time to form a complex structure. The aggregation process is not random [15]; instead it appears to be a repeatable sequential process mediated by bacterial adhesins that allow organisms to aggregate to surfaces and other bacteria by binding to specific receptor moieties (Fig. 1A). The initial colonizers are capable of binding to salivary pellicle receptors, and the subsequent organisms proceed to bind to the initial colonizers [12], [14], [16]. Late colonizers, such as *P. gingivalis,* which has been linked to periodontal disease, are found in the final layer of this complex structure [17]. Some steps of the colonization process can lead to mutual exclusion between closely related species (e.g. streptococci), leading to drastically different macroscopic disease-related outcomes [18].

In this work we test the hypothesis that metabolism is a predictor, and potentially a major driving force, of the order of colonization in the oral biofilm. Specifically, based on the individual inter-species interactions mapped by Kolenbrander ([12] [19], Fig. 1) we quantify the overlap in metabolic functions between adjacent organisms, and compare the distribution of such overlaps to the one obtained for randomized biofilms or for random assemblages of bacteria. We find that the real biofilm is characterized by a significantly larger overlap in metabolic functions between adjacent species, relative to randomized biofilm compositions and structures. Specific metabolic pathways can be associated with the different layers, providing a snapshot of the gradient of metabolic requirements across the biofilm. The observed tendency towards maximal metabolic overlap is likely counteracted by an opposite trend driven by the synergistic advantage of combining the metabolic capabilities of sufficiently different species. In all, these findings suggest that an optimal tradeoff between resource sharing and functional synergy may constitute a fundamental property of structured microbial communities. Our approach, much more detailed than broad functional enrichment studies, but much less demanding than stoichiometric flux balance models, should be broadly applicable to other microbial ecosystems, where spatial or temporal order matters.

## Results

### Metabolic Proximity among Different Layers in the Oral Biofilm

We ask whether the order of colonization in the human dental biofilm may reflect a quantitative principle of microbial ecosystem organization. While the structure of the biofilm from the Kolenbrander model (Fig. 1A, and [12]) reflects known mutual binding between adjacent species, we develop our analysis based on the premise that such binding effects reflect fundamental adaptations to environmental gradients and mutual metabolic exchange between species. Hence, we analyze the biofilm structure in terms of mutual distances between adjacent organisms. 16S rRNA-based distances are standard practice when estimating the similarity of organisms without dealing with the complexities of whole genome alignment [3], [10]. The assumption that 16S rRNA is conserved allows investigators to ascertain evolutionary relationships between organisms. Here, however, we wish to utilize a metabolic, rather than an evolutionary distance [17] (See Methods). Such a metabolic distance will provide a method to gauge the difference in biochemical functions between different species. It is important to note that a metabolic metric can be used to compare biochemical abilities of different organisms without constructing full-fledged genome-scale stoichiometric models, such as the ones built for several microbial species [20]–[26], including the oral pathogen *P. gingivalis* [17]. We expect that organisms with similar enzyme profiles (based on the above metric) will have a comparable ability to utilize and process metabolites from their environment. The specific metabolic distance we use in this work is a standard metric (Jaccard’s distance, *J*) gauging the degree of dissimilarity between the sets of enzymes present in the two organisms (see Methods).

**Figure 2:**
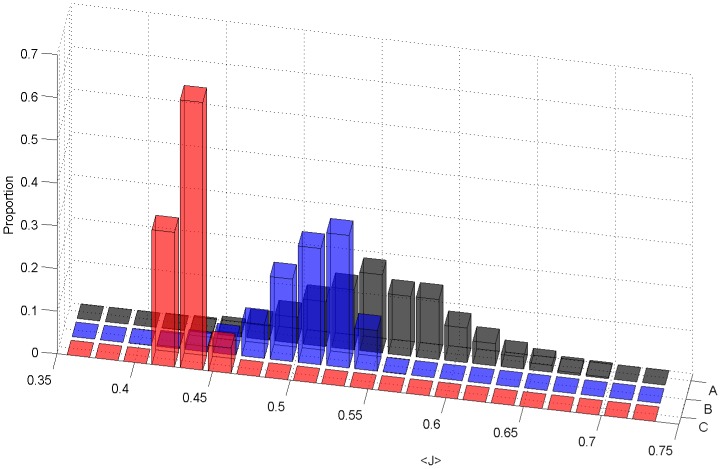
Metabolic distance distributions for correct and randomized orders of colonization. Distributions of average pairwise Jaccard’s distance are compared across different computational realizations of the 11-species biofilm. In particular, we show in red (C) the distribution of average pairwise distances between the 11 organisms for all paths that reflect the layered structure of the Kolenbrander map (see Fig. 1B). In blue (B) we show the distribution obtained for all possible random orders that do not necessarily reflect the layered order of colonization (e.g. path shown in Fig. 1C). The last distribution (grey, A) is obtained from choosing in random order 11 random prokaryotes from the KEGG database.

As a first step towards ascertaining the validity of this premise, one can test whether the 11 organisms that are in the dental biofilm are on average closer to each other than 11 randomly chosen prokaryotes from the KEGG database [27]–[30]. To this end, 1000 random groups of 11 organisms were chosen from the list of KEGG prokaryotes and the sum of pairwise metabolic distances were calculated for each permutation (11 factorial potential orders) for a given random group. The average of all permutation scores was calculated for each random group. We found that the groups of 11 organisms belonging to the oral biofilm model tend to have a smaller average of pairwise metabolic distances (*J*) than 1000 randomly selected groups of prokaryotes (Fig. 2). This is not unexpected, as the organisms inhabit the same niche in the human body and hence must have some underlying metabolic similarity to cope with the common environment. It is interesting to note that the mean distance value between organisms of the dental biofilm is far from being close to a global minimum, when compared to its position in the distribution. This could be due to the fact that organisms with similar metabolic requirements will compete for the same environmental niche and probably would not be found in close proximity to each other within a complex multi-species biofilm.

The next question we ask is whether a special pattern of inter-species metabolic distances can be observed between adjacent organisms in the layered structure of the oral biofilm. In Fig. 1A we present a simplified version of the model presented in [12]. The simplified model contains only organisms whose genome has been sequenced, and whose annotation is available in KEGG. We translate the Kolenbrander map into a set of possible orders of colonization by assuming that an organism can join the biofilm only if it can bind to an organism that that is already present in the biofilm, or to an environmental anchor point. In this way, we determine 864 orders in which the bacteria may join, consistently with the reduced Kolenbrander model (Fig. 1B). The 864 orders (3!×3!×4!) come from all permutations that allow organisms to be placed in their correct layers. Conversely, there would be 39,916,800 (i.e. 11!) orders which disregard the network of experimentally known interactions that constitute the layered model (Fig. 1A). For each given order of colonization, we compute the average Jaccard’s distance between metabolic compositions of adjacent organisms (<*J*>, see Methods). Fig. 2 shows that the distribution of average distances for the orders of colonization compatible with the Kolenbrander model is markedly shifted to the left relative to the background distribution of random orders (P<2·10^−7^). This implies that spatially adjacent organisms in the biofilm have a larger number of common metabolic enzymes relative to randomly chosen pairs. In order to ascertain the overall robustness of this result we repeated the analysis upon different types of perturbations. In particular, we tried to omit from the calculation the *Streptococci* species, which are phylogenetically and metabolically very close to each other, and might therefore bias the result towards high significance. Despite removing these organisms, we still found a statistically significant p-value (P<1.2·10^−4^). Furthermore we verified that the results are not too sensitive to removal of specific enzymes. In fact, we found that the result is still significant when up to 60% of the enzymes used in the analysis are removed (Fig. S1). The “minimal metabolic distance” criterion apparently satisfied by the non-random orders of colonization may be indicative of the way dental biofilm is thought to form. Each group of organisms creates a micro-environment that is conducive for the next set of organisms. Each new organism joining the biofilm can take advantage of the micro-environment generated by organisms that precede it, provided that the inter-species metabolic distance is small enough to make this build-up favorable.

**Figure 3:**
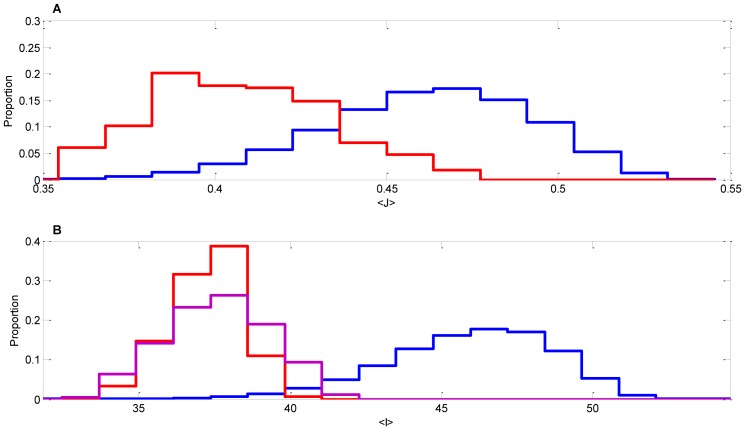
Distributions of alternative metrics for correct and randomized orders of colonization. (A) Similar to what shown in Fig. 1B and Fig. 2, we computed inter-species distance between organisms along paths that respect (red) or do not respect (blue) the layered order of colonization of the Kolenbrander map. Here, however, as opposed to Fig. 2, we compute the Jaccard distance between two species based on their profiles of non-enzyme genes (as identifiable through KEGG KO numbers). (B) The correct and incorrect orders of colonization are compared based on an information metric, rather than on the Jaccard distance. In walking along a colonization order path from one organism to the next, we compute (in Nats) the amount of information added due to the presence of previously absent enzymes. The added information for each pair of adjacent organisms is summed to form the added information score, along paths that respect (red) or do not respect (blue) the layered order of colonization. The purple distribution is obtained by computing the added information scores for orders of colonization that reflect the layered structure, but walk through it in reverse order (i.e. from the outer layer downwards towards the salivary pellicle).

The above analysis demonstrates that adjacent species in the correct order of colonization tend to minimize their mutual metabolic enzyme distance. This finding does not rule out the possibility that similar effects might be observable based on alternative metrics that take into account other (non-metabolic) gene categories. In other words, is the order of colonization pattern reported predominantly a metabolic effect, or does it reflect a general inter-species distance? To address this question, we performed the same analysis shown in Fig. 2 for a large set of non-metabolic genes (see Methods). As shown in Fig. 3A, the correct and random order distributions are still significantly different (P<0.0023), but their separation is much less dramatic than what is found with metabolic enzymes (P<2·10^−7^). Given this result, we can infer that metabolism is one of the most important factors in oral biofilm organization, more so than other classes of genes as a whole.

Finally, to confirm that the pattern observed is not strongly dependent on the metric used, we verified that an alternative, widely used metric can discriminate between correct vs. randomized order of colonization. In particular, we calculated the amount of Shannon information that each organisms adds to the system relative to the previous organism in the order of colonization (see Methods). As with the distance calculation, the overall score is the sum of the pairwise added information values for a given order of colonization. Using a 2-sample Komogrov-Smirnov test [31], we found that literature-informed orders are significantly (P<4.5·10^−6^) smaller than randomized distributions (Fig. 3B). An interesting aspect of the information content metric is that, in contrast to the metabolic distance defined above, it can also capture directionality, i.e. discriminate between a colonization order that starts with the *Streptococci* layer (the first, pellicle-bound layer of the biofilm, Fig. 1A) and one that ends with *Streptococci* layer.

**Figure 4:**
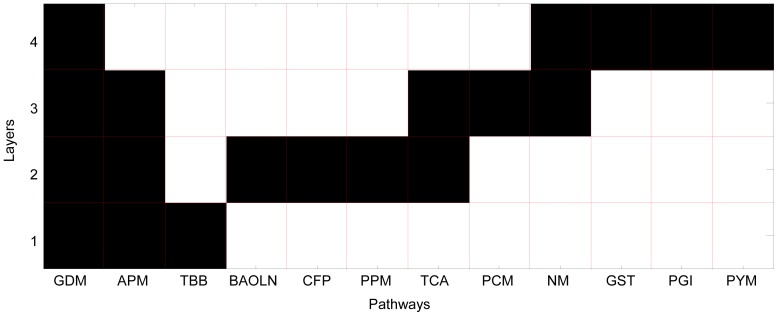
Metabolic pathway enrichment across layers. Based on the enzyme content of the different species found in different layers of the biofilm (with layers labeled from 1 to 4, see Fig. 1), one can estimate whether any given layer is enriched for specific metabolic functions. Enzyme and pathway enrichments for each layer are computed based on a standard GSEA algorithm. Black boxes in the pathways-by-layers matrix denote enrichment of a particular KEGG pathway in a given layer. The pathway abbreviations are as follows: APM-Arginine and proline metabolism, BAOLN-Biosynthesis of alkaloids derived from ornithine lysine and nicotinic acid, CFP-Carbon fixation pathways in prokaryotes, GDM-Glyoxylate and dicarboxylate metabolism, GST-Glycine, serine and threonine metabolism, NM-Nitrogen metabolism, PCM-Porphyrin and chlorophyll metabolism, PGI-Pentose and glucuronate interconversions, PPM-Propanoate metabolism, PYM-Pyruvate metabolism, TBB-Terpenoid backbone biosynthesis, and TCA-Tricarboxylic acid cycle.

If, as suggested by the above results, metabolism is a fundamental determinant of the order of colonization, we would expect to be able to find that specific metabolic pathways can be associated with different layers of the biofilm. Indeed, by performing a GSEA (Gene Set Enrichment Analysis) for metabolic functions (see Methods and Dataset S1), we found that gradients of metabolic functionalities span the different layers (Fig. 4). The pathways that displayed significant enrichment are: arginine and proline metabolism, biosynthesis of alkaloids, carbon fixation, glyoxylate and dicarboxylate metabolism, glycine, serine and threonine metabolism, nitrogen metabolism, porphyrin and chlorophyll metabolism, pentose and glucuronate interconversions, propanoate metabolism, pyruvate metabolism, terpenoid backbone biosynthesis, and tricarboxylic acid cycle. Enzymes related to carbohydrate and proline metabolism are enriched at the initial colonizer stage, possibly allowing for utilization of available carbohydrates as a source of energy that is present in the saliva [32]. The second biofilm layer contains both propionate and TCA pathways. Both require, as input, compounds such as lactate, a byproduct of carbohydrate metabolism which in turn is converted into cytotoxic byproducts [33]. Additionally, butyrate is known to affect gingival epithelial cells inducing apoptosis in sufficient concentrations [34]. Apoptosis of tissues provides organisms with a highly enriched food source [35]. Fumarase and succinate dehydrogenase, are both enriched in this layer of the biofilm. Both enzymes provide a pathway for proline metabolism byproducts to be funneled into energy production which takes advantage of proline catabolism enrichment in the previous biofilm layer. The third layer of the biofilm is enriched for porphyrin metabolism, a pathway that is essential to *Porphyromonas gingivalis*. The organism has a well-known requirement for heme and has been implicated in a number of disease processes such as chronic periodontitis [36]. It is however not capable of producing heme for itself, and must therefore scavenge it from the environment. Correspondingly, we found enrichment for enzymes related to heme production, in particular along a pathway that converts L-glutamate to 5-aminolevulinate, a precursor of heme. In the fourth layer of the colonization process, we find enrichment for nitrogen-related and TCA-cycle genes. This specific combination could reflect amino acids from tissue degradation being shunted into cellular metabolism via entry points within the TCA cycle.

### Determination of Optimal Metabolic Overlap Using Flux Modes

The above analysis of metabolic distances between adjacent organisms in the oral biofilm demonstrates that the correct order of colonization displays close to minimal average metabolic distance relative to randomized orders. Another way of formulating this principle is that, within a biofilm, organisms next to each other will be as close as possible in terms of their metabolic enzyme content. Taken to the extreme, this principle would suggest that biofilms may be preferably composed of rather similar and uniform species (compatibly with the biofilm size and environmental gradients). This is likely not the case, both because organisms metabolically too close to each other may engage in fierce competition for survival [37], and because there may be a physiological advantage to pairing organisms that are neither too close nor too distant from each other.

To formulate a specific hypothesis about this last scenario, we evaluated the metabolic potential of conjoined metabolic networks as a function of their metabolic distance, using elementary flux modes. Elementary flux modes analysis identifies all minimal non-zero flows through a metabolic network [38]. At a first approximation, the number of elementary flux modes can be thought of as an estimate of the size of the space of possible paths through a metabolic network. Here, we sought to estimate the increase in the number of such paths for a pair of interacting metabolic networks (e.g. two bacterial species), relative to the metabolic capabilities of isolated networks, as a function of the similarity between the two networks. The intuition is that when two networks are very similar to each other, there is little added benefit in combining them with each other. At the opposite extreme, if two networks are too different from each other they will “speak different metabolic languages” and barely be able to build significant synergistic pathways. In between these two extremes, there may be an inter-species metabolic distance that provides a maximal synergistic benefit.

**Figure 5:**
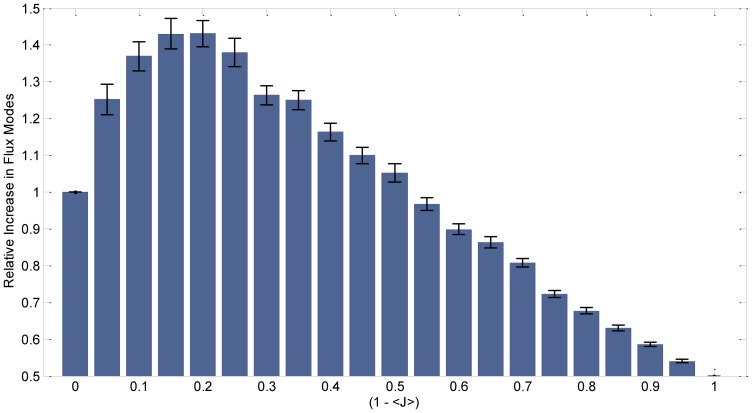
Expected synergy between metabolic networks as a function of metabolic distance. The synergy is computed as the count of elementary flux modes (pathways) that are feasible for a metabolic network that is the union of two networks with a given Jaccard’s distance from each other, normalized to the count of elementary flux modes of the constituent networks. The count of elementary flux modes can be thought of as an estimate of the number of distinct metabolic tasks that the network can perform, i.e. its versatility. Hence, the graph shows how the versatility of two conjoined networks relative to the constituent networks is maximal for an intermediate Jaccard’s distance between such networks. 100 random paired networks were generated for each of several possible Jaccard’s distances. Bar heights reflect the average normalized increase in the number of elementary flux modes, whereas error bars represent the standard error of the mean.

Indeed, upon computing a metabolic synergy score for randomly generated pairs of metabolic networks with a given Jaccard’s distance between each other (see Methods), we found that the mean score displays a distinct profile as a function of inter-network metabolic distance (Fig. 5). In particular, there is a peak at a Jaccard’s coefficient of 0.2. This means that having 33.3% reaction overlap between the component networks is optimal, in the sense that it generates the maximum number of useable balanced pathways (elementary flux modes). The range of Jaccard’s coefficients (i.e. metabolic similarity) within which synergistic interaction is expected extends up to approximately *Jc* = 0.55 (i.e. *J* = 0.45). This means that metabolic networks with a distance below *J* = 0.45 will have little potential for increased biochemical capabilities through metabolic cross-talk. Interestingly, this Jaccard’s distance is very close to the average of the distance among adjacent species in oral biofilm (Fig. 2). A possible interpretation of this result is that the players in the community encounter a tradeoff between maximizing their metabolic overlap, and still not losing the benefit of possible synergistic interactions (i.e. going *beyond J* = 0.45, i.e. *J_C_* = 0.55).

## Discussion

We have addressed the question of whether the spatio-temporal organization of a biofilm can be understood in terms of the differential metabolic properties of individual organisms relative to their neighboring organisms. We implemented a simplified model of the spatial organization of the biofilm, based on experimental evidence of individual pairwise interactions between species. This abstraction of the colonization process enabled us to discretize the order of succession, and systematically investigate all potential permutations of organisms in a linear fashion. A more complex model could more realistically capture inter-species dynamics in physical space [15], without the limitations imposed by taking into account only pairwise interactions and a step-by-step “walk” through the different layers of the biofilm. However, our simplified approach overcomes in an effective way the combinatorial complexity of multi-species networks, and takes advantage of the available pairwise interaction data. We found that metabolic similarity is a highly informative indicator of vicinity in the biofilm. The metabolic structure of the biofilm is reflected in the existence of multiple layers enriched for specific biochemical pathways. This structure lends credence to the idea that each layer contributes to a gradient of metabolic properties, causing environmental modifications that pave the way for subsequent layers of bacteria. We cannot exclude the possibility that the observed effect might be just a result of metabolically similar organisms adapting to environmentally present gradients (e.g. abundance of oxygen). However, the fact that next-to-minimal metabolic distance is significantly associated with binding between organisms in the biofilm suggests this metabolic similarity is truly reflective of particular inter-species interactions. The current work is limited to the eleven organisms of the Kolenbrander map whose annotated sequence was publically available (in KEGG) at the time of the analysis. Future extensions could include additional organisms, benefit from improved genome annotation approaches [39] and gradually move to more mechanistic models of microbe-microbe interactions, such as ecosystem-level flux balance models [40]. In addition, while the current evidence we use for a putative order of colonization is based on a collection of multiple *in vitro* individual pairwise interactions, more comprehensive *in vivo* measurements [19] could in the future be used as a more accurate baseline for testing hypotheses.

Our analysis focused on a specific microbial community in which the order of colonization is manifested both in the chronological sequence of events leading to the full biofilm, as well as in the final spatial architecture of the biofilm itself. We envisage that this analysis could be extended to other microbial ecosystem with a similar spatio-temporal organization, such as biofilms on catheters and medical instruments [41]–[44] or microbial mats in hot springs and desert environments [45], [46]. However, the approach we proposed is not limited to communities with a well-defined or known spatial structure, and could be extended to analyze purely temporal orders of colonization in microbial ecosystems whose biomass is found largely in a planktonic phase, or whose detailed spatial structure is not easily observable (e.g., in the gut microbiome [10], [47]). Metagenomic sequencing projects frequently produce 16S rRNA population composition data, which in a longitudinal study provides us with changes in population composition over time. Combining population data with enzymatic profiles from KEGG would make it possible to test whether metabolic proximity is significantly predictive of temporal species-to-species shifts in an ecosystem.

An interesting outcome of our analysis is the hypothesis that multiple counteracting forces may ultimately determine, at the evolutionary scale, an optimal steady state genomic and spatial configuration of different species in a biofilm. Close metabolic proximity seems to be one desirable criterion for spatial vicinity, motivated by uniformity of environmental conditions, and by multiple chances for metabolic cross-feeding. At the same time, organisms which are metabolically too close to each other would likely compete for common metabolic resources. In addition, as we found in Fig. 5, they would have minimal chance for true synergism, i.e. for the capacity to contribute novel metabolic capabilities to the group as a whole. The specific Jaccard’s distances at which the optimum occurs are likely dependent on the specific topology of the underlying reaction networks utilized, and may not be universal (see Methods). However, in our synergy calculations based on elementary flux modes, we retain a similar degree distribution and reaction topology as would be seen in the bacterial species of the oral biofilm. It will be interesting to explore the possibility that the general shape of the curve observed in Fig. 5 could be derived analytically.

Finally, our finding poses an interesting evolutionary chicken and egg dilemma: did the observed metabolic proximity pattern precede or follow the emergence of specific binding affinities between receptors and ligands across species? On one hand, energy and food-related requirements may be hypothesized to dictate the emergence of a biofilm structure. Subsequent adaptations could have optimized inter-species binding interactions to facilitate the formation of an efficient nutrient and energy flow. Conversely, we cannot rule out the alternative possibility that metabolic proximity may have arisen between organisms with a tendency to bind to each other, e.g. through horizontal gene transfer, or by forcing each other to face specific selective pressures. This may be an interesting challenge for future research, in which the experimental investigation of evolving symbiotic system [48] could be complemented by computational studies of evolutionary rates in genomic sequences [49]–[51].

## Methods

### Parsing an Experimental Map of the Oral Biofilm Structure

The present analysis uses the biofilm organization map that is presented in [12], which is based on the collection of several individual experimental papers. This model (to which we will refer as the Kolenbrander model) contains 22 organisms, 11 of which were sequenced and annotated [27]–[30] at the time of our analysis. The 11 organisms for which data is available are listed in Fig. 1A. The map also includes known connections between some organisms and the salivary pellicle, from which we assume that the biofilm starts developing. In the Kolenbrander model, reproduced in simplified form in Fig. 1A, nodes correspond to species (with the exception of *Veionella* and *Eubacterium*, which were reported only at the genus level). Organisms for which there was no KEGG data were omitted from our analysis. An edge between two nodes in the network of Fig. 1A denotes a documented capacity of the two corresponding biofilm constituents to bind to each other.

### Calculation of Enzyme Based Distances

The KEGG database contains information that describes the number and type of enzymes present in an organism’s genome [27]–[30]. Each enzymatic function is associated with an Enzyme Commission (EC) number [52]. In this case, we are not interested in the abundance of any particular enzyme or in its substrate/product stoichiometry, but simply in the presence or absence of such enzyme in a given organism’s genome. Given this information, a binary vector ***S^(A)^*** can be defined to describe the enzyme composition for an organism *A*, with component *S^(A)^_i_* = *1* if enzyme *i* is present in organism *A*, and *S^(A)^_j_* = *0* otherwise. We then evaluate the difference between the metabolic profiles of two organisms by computing the Jaccard’s distance *J(A,B)* between ***S^(A)^*** and ***S^(B)^***, defined as follows:
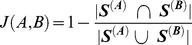



If *A* and *B* have the same metabolic enzymes, then *J* = 0. If they have no enzyme in common, it is *J* = 1. This metric will also be used to quantify metabolic similarity between species, in the form of the Jaccard’s coefficient (*J_c_ = 1−J*) [53].

### Calculation of Non-metabolic Distances

In addition to enzyme content, the KEGG database includes data on the presence of different categories of non-metabolic genes. Using this data we can generate binary vectors ***S^(A)^*** and ***S^(B)^***, that represent the non-metabolic gene content for organisms *A* and *B*, just as was done for metabolic distance. We then evaluate the difference between the non-metabolic profiles of two organisms by computing their Jaccard’s distance *J*(*A,B*).

### Calculation of Added Information

All organisms in the community contribute to an overall super-set of enzymes that represents the metabolic potential of the community. In examining the gradual build-up of the oral biofilm, we can ask how much novelty is introduced by each new organism joining an increasingly complex ecosystem. This can be achieved by calculating the amount of metabolic information added to the current super-set upon introducing a new organism to the biofilm. If we call *N_i_* the number of organisms in which enzyme *i* is present (*N_i_*∈{1, 2, …, *N_organisms_*}, with *N_organisms_* = 11), then the probability to find a given enzymatic function *i* in the whole biofilm is *P_i_* = *N_i_*/*N_organisms_*. For an ordered pair of organisms (*A*,*B*), we can identify the set *K*(*A,B*) of all EC numbers *k* such that *S^(A)^_k_ = 0* and *S^(B)^_k_ = 1*. The information added when organism B is added to organism A is then computed as the Shannon information content of all enzymes that are currently added and were not present in the prior organisms, i.e.:
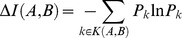



*ΔI(A,B)* represents the amount of Shannon information (relative to the overall abundance of EC numbers in the entire biofilm), added by an organism B upon colonization on top of an organism A. Note that *ΔI* is not symmetric, i.e., in general, *ΔI(A,B)≠ΔI(B,A)*. Hence, this added information metric allows us to distinguish between orders of colonization that would be indistinguishable using the Jaccard’s metric *J* defined above.

### Layer Specific Pathway Enrichment

To determine possible layer-specific metabolic pathway enrichment in the oral biofilm, we first calculate the proportion of organisms in a given layer that contain a given enzyme. This calculation uses the same organism-specific binary vector ***S^(A)^*** defined above. If the set of organisms in biofilm layer *x* is defined as *L_x_*, then a biofilm layer profile (***B^(x)^***), describing the occurrence of each enzyme in any given organism in layer *x* can be calculated as follows:
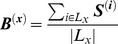



These profiles can be used to estimate the enrichment of the different layers for specific metabolic processes. This is achieved by using gene set enrichment analysis (GSEA). In addition to the ***B^(x)^*** profiles for the four different layers (Fig. 1A), the GSEA algorithm utilizes a binary mapping matrix *K_(i,j)_*, where *K_(i,j)_* = 1 if enzyme *i* is present in pathway *j*. This matrix maps enzymes to corresponding KEGG metabolic pathways. We next look for specific enzyme and pathway enrichment in a given layer relative to other layers within the dental biofilm. The enrichment calculation is performed using a standard GSEA application [54]. Pathways with a nominal p-value of 0.05 or less and an FDR of less than 0.25 were chosen (with FDR accounting for multiple testing biases).

### Construction of Randomized Paired Networks

In order to estimate the metabolic benefit derived from the cooperation of two species, we perform an analysis of elementary flux modes in appropriately modified versions of the *E. coli* FBA model [21]. In particular, we used the *E. coli* FBA model (cytoplasm reactions only) as an initial main network (of size *R_TOT_*) to generate random metabolic networks with a degree distribution and topology similar to that of real metabolic networks. Random networks are generated in pairs, with a specified metabolic similarity (Jaccard’s coefficient, *J_c_*) between them. The algorithmic pipeline to generate such pairs of networks proceeds as follows:

- Out of the main source network of size *R_TOT_*, we choose a subnetwork (the “source network”) of size 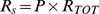, where *P* is a given percent coverage, chosen in order to guarantee tractability of the elementary flux modes calculations. The standard value used throughout this work is P = 0.8.
- Given the desired degree of metabolic overlap (*J_c_*) between the two networks, and the size of the source network (*R_s_*), we compute the size *R_int_* of the set *N^int^* of reactions that should be in common between the two networks. This number is simply

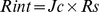

- We select two random sets (*N^1^, N^2^*) of non-overlapping reactions from the source network. Each of these reaction sets is chosen to have size
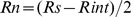

- We use the sets of reactions *N^int^*, *N^1^* and *N^2^* to build reaction sets for the two desired randomized networks, and for their union. These reaction sets for individual networks (*C^(1)^*, *C^(2)^*) and for their joint combination (*C^(1,2)^*) are defined as follows:

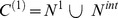


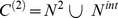


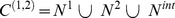

- The reaction sets *C^(1)^ C^(2)^* and *C^(1,2)^* are mapped to stoichiometric matrices *M^1^* and *M^2^* and *M^(1,2)^* respectively. When generating the combined stoichiometric matrix *M^(1,2)^*, we need to make sure that no additional overlap (in addition to the chosen *N^int^* reactions) is introduced between the two random networks. This is achieved by ascribing new names (i.e. new stoichiometric matrix rows) to the metabolites contributed by *N^1^* and *N^2^* which are not already present in *N^int^*.

### Computation of Degree of Metabolic Synergy Using Elementary Flux Modes

For the stoichiometric matrices described in the previous paragraph, the number of elementary flux modes (EFM) is calculated using the *efmtool* software [38]. For each stoichiometric matrix passed to it, *efmtool* returns an EFM matrix describing the various elementary modes through the network. The number of columns of the matrix corresponds to the number of EFMs possible for the given network. A normalized score estimating the increase in the number of EFMs obtained upon conjoining two networks can be computed as follows:
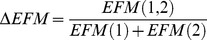



Where *EFM*(1,2) is the number of elementary flux modes from the joint network as defined by *M^(1,2)^*, while *EFM*(1) and *EFM*(2) represent the numbers of elementary flux modes generated by the constituent randomized stoichiometric matrices *M^1^* and *M^2^* respectively. *ΔEFM* represents the increase in the number of flux modes, relative to the constituent networks.

## Supporting Information

Figure S1 **Sensitivity analysis of our metabolic approach for recapitulating the order of colonization, upon gradual removal of information.** The distributions of pairwise metabolic distances for correct (literature-informed) and randomized orders of colonization are plotted for different percentages of enzymes removed from the dataset. Between 20 and 80 percent of enzymes were removed.
(PDF)

Dataset S1 **This file contains tables listing the presence and absence of different KEGG metabolic pathways within the 11 organisms used in this study.**
(XLSX)
