# Supplementary figures and images for: Metabolic Proximity in the Order of Colonization of a Microbial Community
Source: PLoS One. 2013 Oct 30;8(10):e77617. doi: 10.1371/journal.pone.0077617 (PMC3813667; doi:10.1371/journal.pone.0077617)

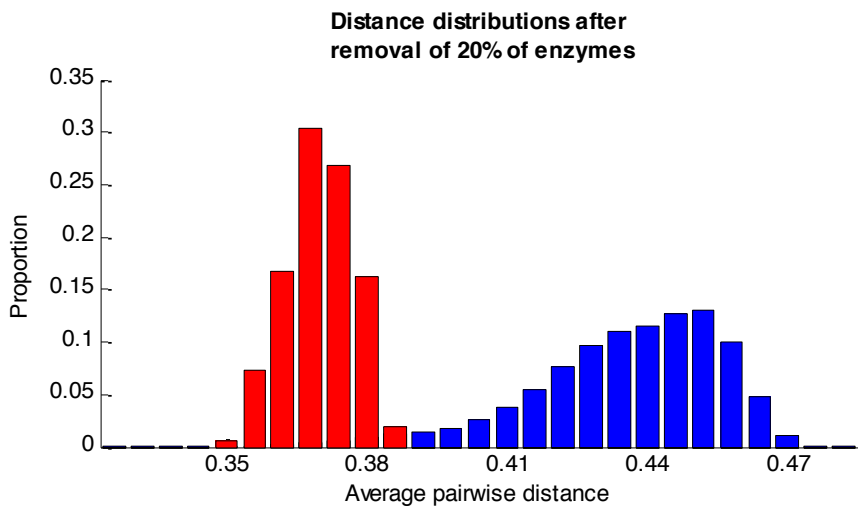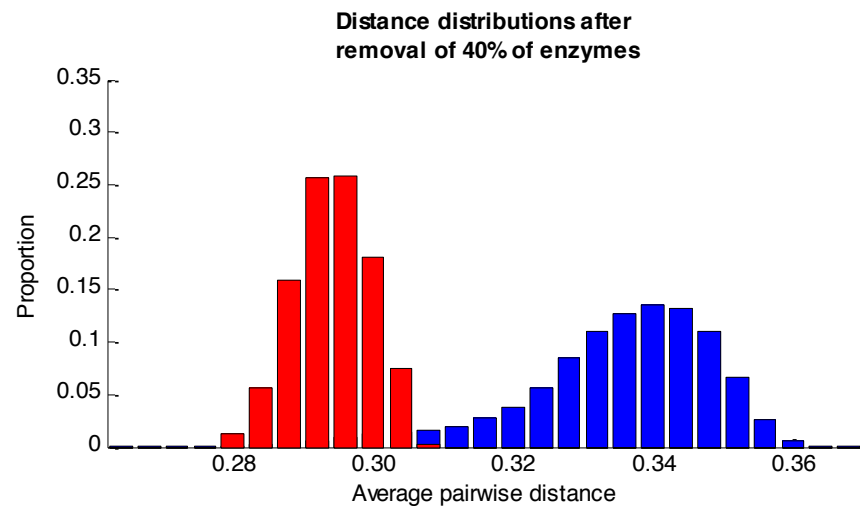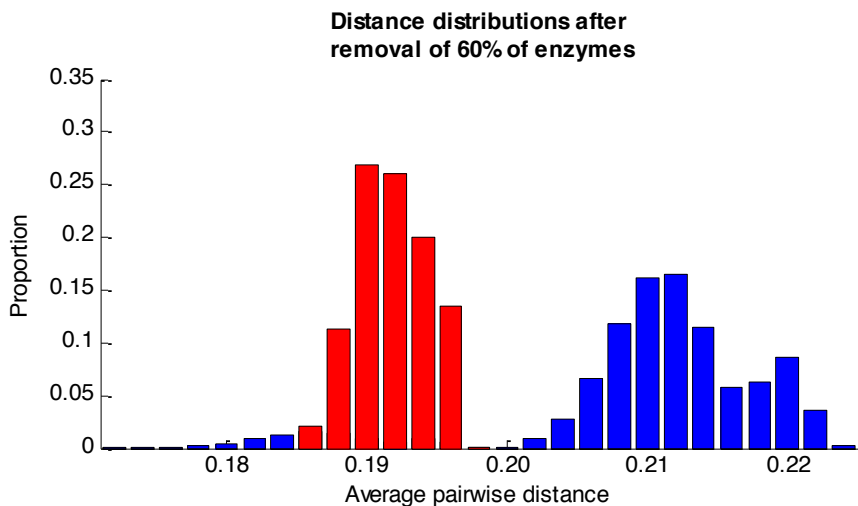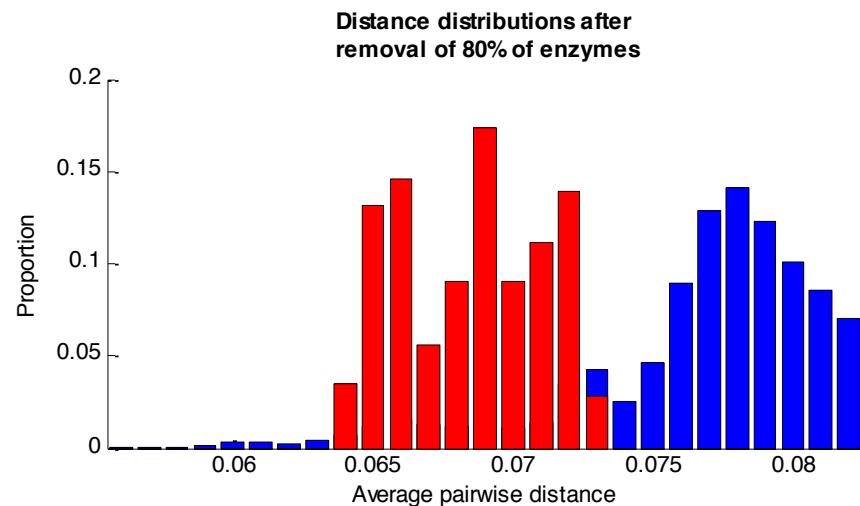

Supplement: Figure S1 — Sensitivity analysis of our metabolic approach for recapitulating the order of colonization, upon gradual removal of information. The distributions of pairwise metabolic distances for correct (literature-informed) and randomized orders of colonization are plotted for different percentages of enzymes removed from the dataset. Between 20 and 80 percent of enzymes were removed. (PDF) [file pone.0077617.s001.pdf]
